# Brain and spine MRI findings in children presenting with TMCO1 mutation

**DOI:** 10.1259/bjrcr.20210253

**Published:** 2022-06-08

**Authors:** Charith Ratnayake, Srikala Narayanan, Jenna Gaesser, Subramanian Subramanian

**Affiliations:** 1 University of Pittsburgh School of Medicine, Pittsburgh, Pennsylvania; 2 Division of Pediatric Radiology, UPMC Children’s Hospital of Pittsburgh, Pittsburgh, Pennsylvania; 3 Department of Radiology, UPMC Children’s Hospital of Pittsburgh, Pittsburgh, Pennsylvania; 4 Department of Pediatrics, Child Neurology, UPMC Children’s Hospital of Pittsburgh, Pittsburgh, Pennsylvania

## Abstract

Cerebrofaciothoracic dysplasia (CFTD) is a developmental disorder characterized by distinctive craniofacial dysmorphism, global developmental delay, and skeletal anomalies. CTFD is the result of biallelic autosomal recessive loss of function mutations in the transmembrane and coiled-coil domains one protein (TMCO1) gene. Based on a population of 27 molecularly confirmed cases, classic brain morphologies associated with CFTD have been described in the literature. Previous studies have demonstrated only mild ventriculomegaly, corpus callosum abnormalities, frontotemporal atrophy, and three cases of associated epilepsy. We present previously undescribed brain MRI findings in two children presenting with seizures due to TMCO1 mutation. MR Imaging demonstrated hippocampal malrotation, olfactory bulb agenesis and olfactory sulcus hypoplasia in both children, pontine hypoplasia, and cochlear nerve agenesis in one child. We demonstrate that TMCO1 may play a more extensive and previously undescribed role in neurodevelopment thereby expanding the phenotype associated with CFTD.

## Introduction

CFTD was initially described by Pascual-Castroviego et al^1^ through observational studies in consanguineous Old Order Amish communities.^2^ They described three patients presenting with characteristic findings of mental retardation, narrow forehead, hypertelorism, broad nose, low hairline, brachycephaly, multiple bony abnormalities in the upper thoracic vertebrae and sometimes in cervical region, among others. In 2010, Xin et al^3^ identified a novel autosomal recessive condition in a group of 11 undiagnosed patients in an Amish community in northeastern Ohio with similar phenotypes. They were able to detect a homozygous deletion in the transmembrane and coiled-coil domains one protein (TMCO1) gene that lead to a severely truncated protein product, connecting the TMCO1 genetic variant with CFTD.

As subsequent studies have described additional patient populations with the characteristic genetic mutation, the phenotype of CTFD has also expanded. Prenatal, craniofacial, oral/dental, skeletal, psychosocial, and neurologic manifestations have all been described. Notably, brain imaging findings include ventricle abnormalities, corpus callosum dysgenesis, cerebellar abnormalities, septum pellucidum cysts, and myelination defects.^4^

### Case 1

**Figure 1.**
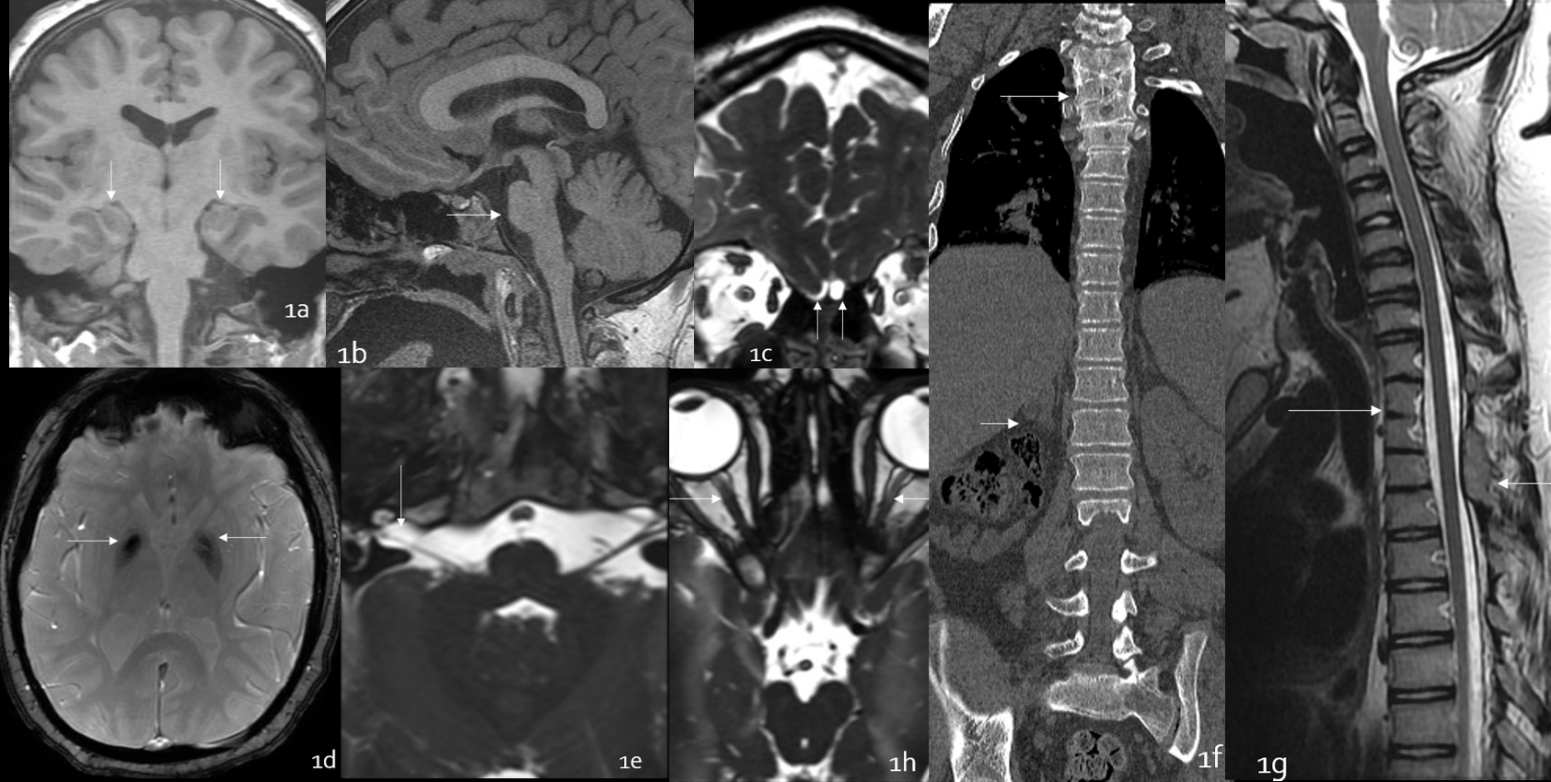
15-year-old male with TMCO1 mutation presenting with seizures. Coronal T1 (1a) demonstrates bilateral hippocampal malrotation (arrow); sagittal T1 FSPGR (1b) demonstrates pontine hypoplasia (arrow) ; coronal fast imaging employing steady state acquisition sequence (1c) demonstrates absence of bilateral olfactory bulbs and hypoplastic olfactory sulci (arrow); axial SWI sequence (1d) demonstrates abnormal susceptibility in globi pallidi (arrow) and axial fast imaging employing steady state acquisition sequence (1e) demonstrates absent right cochlear nerve (arrow); coronal CT bone window reformats (1f) and sagittal T2 (1g) demonstrates thoracic vertebral segmentation abnormality (long arrow), normal spinal cord and absent right kidney (short arrow). Fast imaging employing steady state acquisition sequence demonstrates hypoplastic bilateral optic nerves (1 h). FSPGR, fast spoiled gradient-echo; SWI, susceptibility-weighted imaging; TMCO1, transmembrane and coiled-coil domains one protein.

A 15-year-old Amish male with history of uncontrolled epilepsy (seizures since 3 years of age), cognitive impairment, and abnormal leg movements for 4 years presented with whole body tremors, eye rolling, loss of consciousness, staring and unresponsiveness. His milestones were delayed, and he started to walk at the age of 4 and had repeated history of falls, resulting in one episode of intracranial hemorrhage and poor balance. He has craniofacial dysmorphism including squired face, brachycephaly, synophrys, and wide set eyes. He had thoracic vertebral segmentation and rib fusion anomalies. His genetic testing was consistent with biallelic autosomal recessive TMCO1 mutation. Family history is significant for two sisters with TMCO1 mutation. He was admitted and had an EEG which demonstrated epileptiform discharges in the form of right temporal slow waves, generalized background slowing and right temporal polymorphic δ activity. His physical examination demonstrated no focal neurological findings. He had 2+ patellar reflex and biceps reflex. His gait could not be assessed as he was wheelchair bound. An MRI brain and entire spine was obtained which revealed bilateral hippocampal malrotation (Figure 1a), mild pontine hypoplasia (Figure 1b), absent olfactory bulb and tracts and hypoplastic olfactory sulci (Figure 1c). There was prominent mineralization in bilateral globi pallidi (Figure 1d). In addition, the patient also had right cochlear nerve agenesis (Figure 1e). In order to further evaluate the thoracic spine segmentation, abnormality CT and MRI was obtained which demonstrated segmentation abnormalities in first through fifth thoracic vertebrae and fusion of posterior elements of third through fifth thoracic vertebrae and seventh cervical and first thoracic vertebrae (1f &1g); however, the spinal cord was normal (Figure 1g). There was right renal agenesis (Figure 1f). There was bilateral optic nerve hypoplasia (1h). Congenital rib anomalies were also noted. The patient was treated with carbamazepine for epilepsy, and he is undergoing physical therapy.

### Case 2

**Figure 2.**
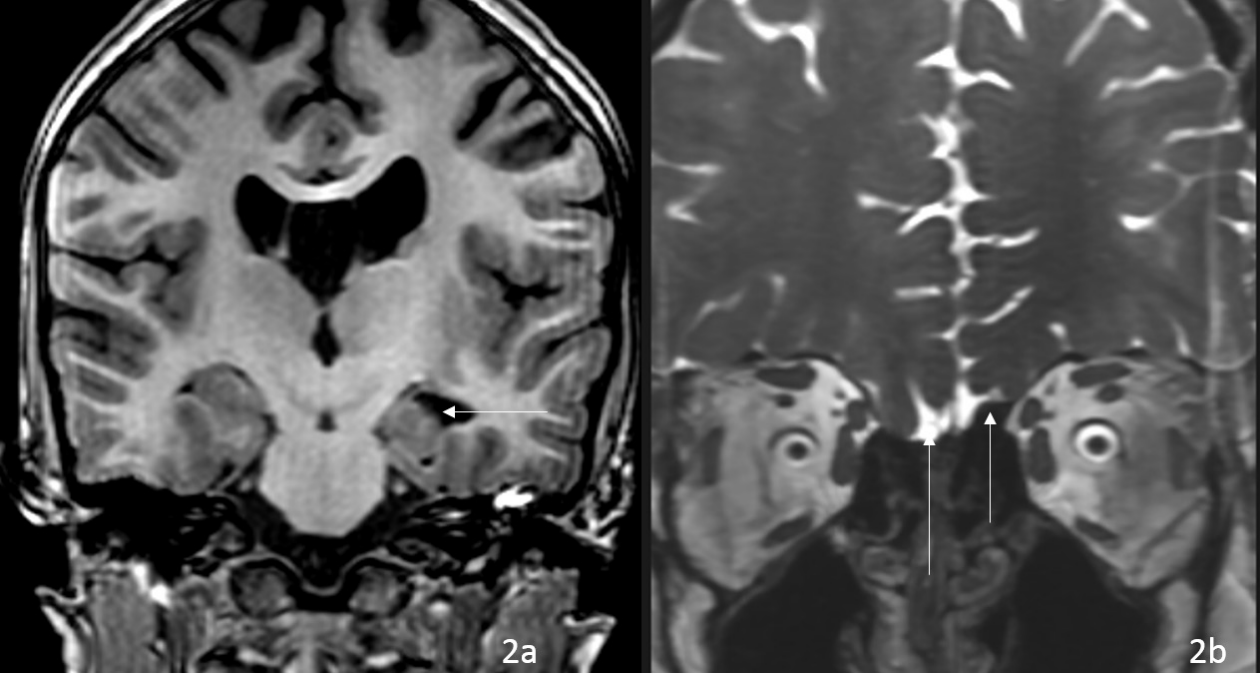
9-year-old female with TMCO1 mutation presenting with epilepsy and MRI demonstrates left hippocampal malrotation (arrow) (2a), absent olfactory bulb (arrow), and hypoplastic bilateral olfactory sulci (2b), bilateral lateral ventriculomegaly and cavum septum pellucidum were present on coronal *T*_1_ weighted imaging and coronal fast imaging employing steady state acquisition sequence, respectively. TMCO1, transmembrane and coiled-coil domains one protein

A 9-year-old female with known homozygous TMCO1 mutation, heterozygous GRIN2A variant of unknown significance, heterozygous variant of unknown significance in KCNH2 and SLC12A5 and seizures presented to UPMC Children’s Hospital of Pittsburgh emergency department with altered mental status after increase in valproic acid 4 weeks before for increasing seizures. She was admitted with suspicion for valproic acid toxicity and continuous EEG was obtained which demonstrated right temporal intermittent slowing admixed with sharp and spike waves without seizures. She was sleepy, but otherwise clinical examination revealed no focal neurological signs. A urine drug test was negative in ED. She was born full term and had developmental delay and a maternal cousin with seizures. A lumbar puncture was normal. HSV PCR, CSF autoimmune panel, and CSF culture were all negative. Laboratory evaluation for valproic acid levels, ammonia, and liver function tests were normal. MRI brain was obtained to exclude encephalitis. MRI demonstrated left hippocampal malrotation (Figure 2a), absent bilateral olfactory bulbs and tracts (Figure 2b), hypoplasia of bilateral olfactory sulci, mild bilateral lateral ventriculomegaly, cavum septum pellucidum and left middle cranial fossa arachnoid cyst. Her seizure treatment was optimized and there was clinical improvement in mental status and patient was discharged.

## Discussion

We report previously undescribed neuroimaging findings in a confirmed case of CFTD, a rare syndrome presenting with characteristic dysmorphic features, psychomotor delays, and neurologic abnormalities. Specifically, both patients had epilepsy, hippocampal malrotation, and olfactory bulb and tract agenesis. Both patients had prominent susceptibility in bilateral globi pallidi, but this was more pronounced in Patient 1, who was also much older than Patient 2. Patient 1 also demonstrated hypoplasia of optic nerves, pontine hypoplasia, and right cochlear nerve agenesis. As such, it is possible that TMCO1 may play a more pronounced role in neurodevelopment than previously described.

The transmembrane and coiled-coil domains one protein directs formation of endoplasmic reticulum calcium leak channels which facilitate calcium leak upon overload of the endoplasmic reticulum.^5^ Failure of calcium leak results in abnormal cell function resulting in increased ER stress leading to delayed osteogenesis, reduced mitochondrial volume and respiration, and upregulation of the unfolded protein response and ER-associated degradation.^5,6^ It is possible that increased calcium deposition may be a reason for prominent susceptibility in the globi pallidi.

Calcium channel signaling is integral to the development of nervous system.^7,8^ Various calcium influx pathways (Nicotinic AChRs, TRP, CRAC, NMDA, AMPA/KA receptors) have been implicated in the proliferation, migration, and differentiation of nascent neurons.^7,8^ Therefore, aberrant calcium channel activity through severe loss of function of calcium leak channels, such as TMCO1, may lead to severe pathology in neurodevelopment. For example, olfactory bulb agenesis may be due to abnormal development of the cribriform plate of the ethmoid and failure of olfactory nerves to induce development of the olfactory bulb from the telencephalon. Similarly, hippocampal malrotation can also reflect part of a larger spectrum of underlying cerebral malformations. In addition, we identified other midline abnormalities like optic nerve and optic chiasm hypoplasia.

CFTD remains a rare developmental disorder with a wide and varied phenotypic presentation. We describe additional neuroimaging findings which will contribute to expanding the neuroimaging phenotype of patients with CFTD.

## Learning points

- TMCO1 mutation leads to abnormal brain development including absent olfactory bulbs and tracts, hippocampal malrotation, midline abnormalities including optic nerve hypoplasia and pontine hypoplasia in addition to previously described ventriculomegaly, corpus callosum thinning, frontotemporal atrophy, vertebral segmentation and rib abnormalities.
- Epilepsy is uncommon in children with TMCO1 mutation, however, present in both children in present report.
